# Prevalence, Antimicrobial Resistance, Virulence Genes and Genetic Diversity of *Salmonella* Isolated from Retail Duck Meat in Southern China

**DOI:** 10.3390/microorganisms8030444

**Published:** 2020-03-21

**Authors:** Zhengquan Chen, Jie Bai, Shaojun Wang, Xibin Zhang, Zeqiang Zhan, Haiyan Shen, Hongxia Zhang, Junping Wen, Yuan Gao, Ming Liao, Jianmin Zhang

**Affiliations:** 1 National and Regional Joint Engineering Laboratory for Medicament of Zoonoses Prevention and Control, Guangdong Laboratory for Lingnan Modern Agriculture, Key Laboratory of Zoonoses, Ministry of Agriculture, Key Laboratory of Zoonoses Prevention and Control of Guangdong Province, College of Veterinary Medicine, South China Agricultural University, Guangzhou 510642, China; 2 Lab of Beef Processing and Quality Control, College of Food Science and Engineering, Shandong Agricultural University, Taian 271018, China; 3 New Hope Liuhe Co., Ltd., Beijing 100102, China; 4 Scientific Observation and Experiment Station of Veterinary Drugs and Diagnostic Techniques of Guangdong Province, Ministry of Agriculture, Key Laboratory of Livestock Disease Prevention of Guangdong Province, Institute of Animal Health, Guangdong Academy of Agricultural Sciences, Guangzhou 510640, China

**Keywords:** retail markets, duck meat, *Salmonella*, antimicrobial resistance, virulence genes, pulsed-field gel electrophoresis

## Abstract

*Salmonella* is an important cause of foodborne diseases. This study was undertaken to investigate the prevalence, serotype distribution, antimicrobial resistance, virulence genes, and genetic diversity of *Salmonella* isolates recovered from fresh duck meat obtained from retail markets in Southern China. In total, 365 samples of fresh duck meat were collected from retail markets in six different cities of Guangdong Province between May 2017 and April 2019. High levels of *Salmonella* contamination were detected in duck meat (151/365, 41.4%). Twenty-six different *Salmonella* serotypes were identified: *S.* Corvallis (*n* = 25, 16.6%), *S.* Kentucky (*n* = 22, 14.6%) and *S*. Agona (*n* = 20, 13.3%) were the most prevalent serotypes. All isolates were resistant to at least one antibiotic and 133 (88.1%) isolates exhibited multidrug resistance (MDR). Most (86.1%) *Salmonella* isolates carried seven classes of virulence-associated genes. This study showed the diversity of *Salmonella* serotypes and genotypes and the high prevalence of MDR isolates carrying multiple virulence-associated genes among isolates from duck meat obtained from retail markets in Southern China. Isolates from different districts had similar pulsed-field gel electrophoresis (PFGE) patterns indicating that circulating foodborne *Salmonella* constitutes a potential public health issue across different districts.

## 1. Introduction

*Salmonella* is an important cause of foodborne diseases associated with increased morbidity and mortality worldwide [1,2]. To date, more than 2600 *Salmonella* serotypes have been reported [3,4]. *Salmonella* infections are the second leading cause of bacterial foodborne illness in the United States [5] and in China, *Salmonella* is the cause of approximately 22.2% of foodborne diseases [6]. Moreover, human *Salmonella* infections result from eating contaminated animal-derived foods, with duck meat recognized as a likely reservoir for *Salmonella* [7,8,9].

Monitoring the presence of foodborne pathogens is a key prerequisite to identify potential problems in food production, processing, preparation, or the sales process proving [8]. Duck meat is an important meat product commonly consumed over the world, such as ducks in Penang Malaysia and Pekin ducks in South Korea [10,11,12,13,14]. According to Food and Agriculture Organization (FAO) data (2017), China is the world’s largest producer and consumer of cultivated waterfowl. In 2017, global duck meat production reached 4.46 million tons, with China accounting for 68.8 % of the total (FAO, 2017). Outbreaks of salmonellosis are related to the consumption of contaminated duck meat, often resulting in serious illness, that may require hospitalization, or even death [11,15]. *Salmonella* contamination of animal-derived foods is particularly serious in China [9,16,17,18,19]. However, there are few reports of *Salmonella* contamination of duck meat worldwide [7,10,14]. Therefore, *Salmonella* contamination of retail duck meat should be addressed through ongoing monitoring and control measures.

Antibiotics play an important role in the treatment and control of salmonellosis, and antibiotic-resistant *Salmonella* has received worldwide attention [20,21]. MDR *Salmonella*-contaminated food is a major global public health concern [22,23,24]. Although several studies of MDR *Salmonella* in animal-derived foods have been reported [8,25,26,27], data related to contamination of duck meat are scarce [7]. Control of MDR *Salmonella* in duck meat through the reasonable use of antibiotics is vital to ensure food safety.

The aim of this study was to investigate the prevalence levels, serotype distribution, antimicrobial resistance, virulence, and genetic diversity of *Salmonella* in retail duck meat in Southern China. This information represents the foundation of follow-up studies on food safety and public health issues caused by *Salmonella*.

## 2. Materials and Methods

### 2.1. Sample Collection

A total of 365 fresh duck meat samples were randomly collected from retail markets in different cities (Guangzhou, Shenzhen, Shaoguan, Foshan, Meizhou, and Zhaoqing) in Guangdong Province between May 2017 and April 2019. A scheme of the sample collection as follows: In Guangzhou city, different regions were sampled monthly; in other cities, samples were taken monthly from three selected retail markets. Samples were taken of different duck types (i.e., whole ducks and chopped ducks) and during different seasons (i.e., spring, summer, autumn and winter). Each sample was marked, placed in a sterile plastic sample bag, transported to the laboratory on ice, and processed immediately.

### 2.2. Salmonella Isolation and Identification

*Salmonella* isolation and identification were performed according to the Standard ISO-6579 (International Organization for Standardization, 2002) method [8,28]. Briefly, 25 g of samples was placed into a sterile plastic bag containing 225 mL of buffered peptone water, shaken for 3 min, and incubated at 36 °C ± 1 °C for 8–18 h. Then 1 mL of the suspension was added to 10 mL each of tetrathionate (TT) broth and Rappaport–Vassiliadis (RV) soya broth and incubated at 42 °C for 18–24 h. After selective enrichment, the suspensions were streaked onto xylose lysine tergitol 4 agar (XLT-4) and CHROMagar^TM^ and incubated at 36 °C ± 1 °C for 18–24 h. Isolates with typical *Salmonella* phenotypes were further confirmed using API 20E test strips (bioMerieux, Marcy-l’Etoile, France). All the *Salmonella* isolates were serotyped according to the White-Kauffmann-Le Minor scheme or by National Food Safety Standard food microbiological examination: *Salmonella* (GB 4789.4-2016) was serotyped by slide agglutination using specific O and H antisera (S&A Reagents Lab. Ltd., Bangkok, Thailand).

### 2.3. Antibiotic Susceptibility Testing

Minimum inhibitory concentrations (MICs) were determined by the agar dilution method using Mueller-Hinton agar according to the standards of the Clinical and Laboratory Standards Institute (CLSI 2013) [29]. A total of 15 antimicrobial agents were tested: AMP (ampicillin), FEP (cefepime), CTX (cefotaxime), IPM (imipenem), NAL (nalidixic acid), CIP (ciprofloxacin), OFX (ofloxacin), STR (streptomycin), GEN (gentamicin), AMK (amikacin), CHL (chloramphenicol), FFC (florfenicol), TET (tetracycline), SUL (sulfadiazine) and PB (polymyxin B). *Escherichia coli* ATCC 25,922 and ATCC 35,218 were used as quality control organisms in MIC determinations. The breakpoints for antimicrobials followed interpretive standards provided by CLSI (2013). Isolates exhibiting resistance to three or more antibiotic classes were defined as MDR *Salmonella*.

### 2.4. Detection of Resistance Genes and Virulence-Associated Genes

All isolates of *Salmonella* were screened for the presence of resistance genes and virulence genes by polymerase chain reaction (PCR). The DNA templates were prepared according to a previously described method [30]. Primers used to amplify the resistance genes in this study are listed in Supplementary Table S1 and virulence-associated genes shown in Supplementary Table S2. The PCR cycling conditions were: 94 °C for 5 min followed by 30 cycles of 94 °C for 30 s, the appropriate annealing temperature for each primer pair for 30 s, and 72 °C for 1 min, with a final extension step of 72 °C for 10 min. The PCR products were analysed by electrophoresis and visualized under ultraviolet light.

### 2.5. Pulse-Field Gel Electrophoresis (PFGE)

PFGE patterns were generated for 151 *Salmonella* isolates according to the protocol developed by the Centers for Disease Control and Prevention (CDC) [31]. Briefly, agarose-embedded DNA was digested with the restriction enzyme *Xba* I (Takara, Tokyo, Japan) for 1·5–2 h in a water bath at 37 °C. Fragments of digested DNA were separated by electrophoresis in 0.5 × Tris-borate-EDTA buffer at 14 °C for 18 h using a CHEF-mapper system (Bio-Rad, Hercules, CA, USA). *S.* Braenderup H9812 was used as the molecular weight standard. PFGE results were analyzed using BioNumerics software version 5.0 (Applied Maths, Sint-Martens-Latem, Belgium).

## 3. Results

### 3.1. Salmonella Prevalence and Serotypes

**Figure 1:**
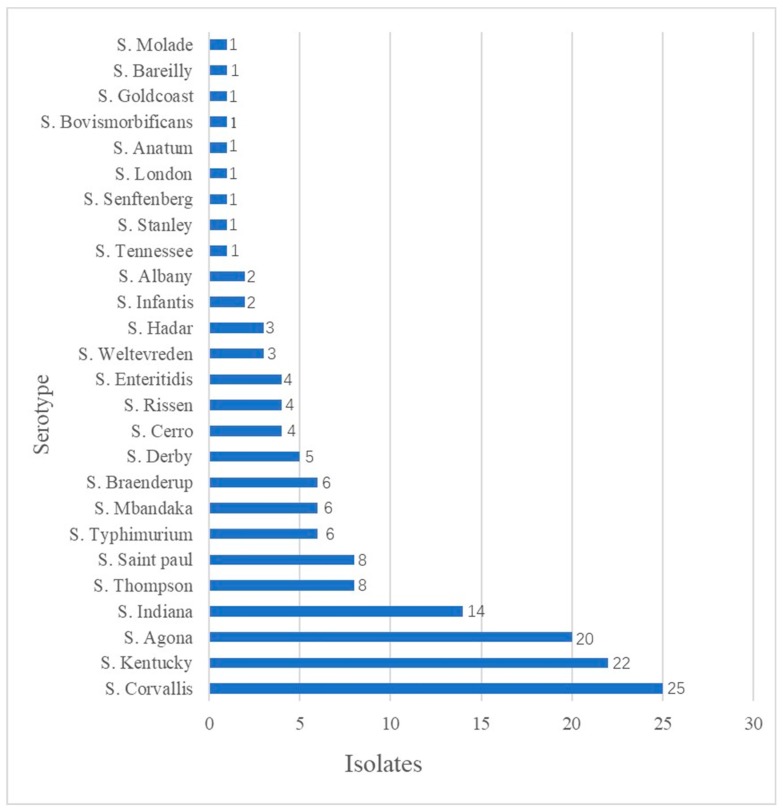
Serotype distribution of *Salmonella* isolates (*n* = 151).

A total of 151 *Salmonella* isolates (151/365, 41.4%) were recovered from the fresh duck meats samples and *Salmonella* was detected during four seasons (Table 1). The most serious contamination occurred during the summer months (62.4%). Twenty-six different serotypes were identified among the *Salmonella* isolates: *S*. Corvallis (25/151,16.6%), *S.* Kentucky (22/151,14.6%) and *S*. Agona (20/151,13.3%) were the dominant serotypes. Moreover, *S.* Bareilly and *S*. Molade were detected in food for the first time in China (Figure 1).

### 3.2. Antibiotic Susceptibility Testing

Antimicrobial resistance phenotypes of 151 *Salmonella* isolates are shown in Table 2. The highest overall levels of resistance were observed for tetracycline (85.4%) and sulfadiazine (84.1%), followed by chloramphenicol (62.3%), florfenicol (60.3%), ofloxacin (56.9%), nalidixic acid (53.6%), streptomycin (45.0%), and gentamicin (31.7%). Lower levels of resistance were found for ampicillin (9.3%) and amikacin (1.3%). However, no isolates were resistant to cefepime, imipenem, or polymyxin B. Importantly, there was a certain degree of resistance to cefotaxime (25/151, 16.6%) and ciprofloxacin (29/151, 19.2%). Among them, 12 *Salmonella* isolates were resistant to both (7 *S*. Kentucky,4 *S*. Indiana and 1 *S*. Agona). All 151 isolates were resistant to at least one antibiotic and 133 (88.1%) isolates were MDR. Resistance to 3–8 antibiotics was detected in 115 isolates (76.2%), 18 isolates (11.9%) were resistant to 9–10 antibiotics, MDR-ACSSuT (7/151, 4.6%), and one *S.* Indiana isolate was resistant to 11 antimicrobials (AMP, CHL, CIP, CTX, FFC, GEN, NAL, OFX, STR, SUL, TET). Notably, MDR was found to be widely distributed in various serotypes of *Salmonella* (Table 3).

### 3.3. Detection of Antimicrobial Resistance Genes

All 151 isolates were selected and examined for antimicrobial resistance genes. Five antimicrobial resistance genes (*bla _SHV_*, *bla _PSE,_ Aaca(3)-Ia*, *qnrA*, and *tet B*) were not detected by PCR in any of the 151 isolates (Table 4). All 34 isolates resistant to the β-lactamase class contained the *bla _TEM_* gene. Two *S*. Agona and one *S*. Kentucky isolates contained the *bla _CTX-M_* gene, but none of the isolates were positive for the *bla _SHV_* and *bla _PSE_* genes. Among the 68 streptomycin-resistant isolates, 64 (94.1%) isolates contained the *strA* gene and 57 (83.8%) isolates had the *aadA1* gene, while none of the isolates were positive for *Aaca(3)-Ia* gene. Among the 81 fluoroquinolones-resistant isolates, 64 (79.0%) isolates had the *qnrS* gene, 21 (25.9%) had the *aac (6′)-Ib* gene and 18 (22.2%) had the *qnrB* gene. Among the 94 chloramphenicol-resistant isolates, 53 (56.4%) isolates had the *floR* gene, although only one *S*. Indiana (1.1%) isolate contained the *cat1* gene.

### 3.4. Detection of Virulence-Associated Genes

All 151 isolates harbored at least one class of virulence-associated gene. All isolates were positive for the SPI_S_ genes *mgtC*, *siiD*, and *sopB*, the enterotoxin gene *stn* and the fimbrial gene *fimA*. Other virulence genes were detected in the *Salmonella* isolates as follows: *ssaQ* (89.4%), *avrA* (86.8%), *spvC* (6.6%), and *spvR* (5.3%). Furthermore, *Salmonella* isolates with different serotypes showed variations in virulence-associated genes (Table 5). For example, most (86.1%) *Salmonella* isolates were found to carry seven classes of virulence genes although only five classes of virulence genes were detected in *S*. Thompson isolates. Nevertheless, eight or nine classes of virulence genes were detected in *S*. Enteritidis, *S*. Typhimurium and *S*. Derby.

### 3.5. PFGE Analysis

**Figure 2:**
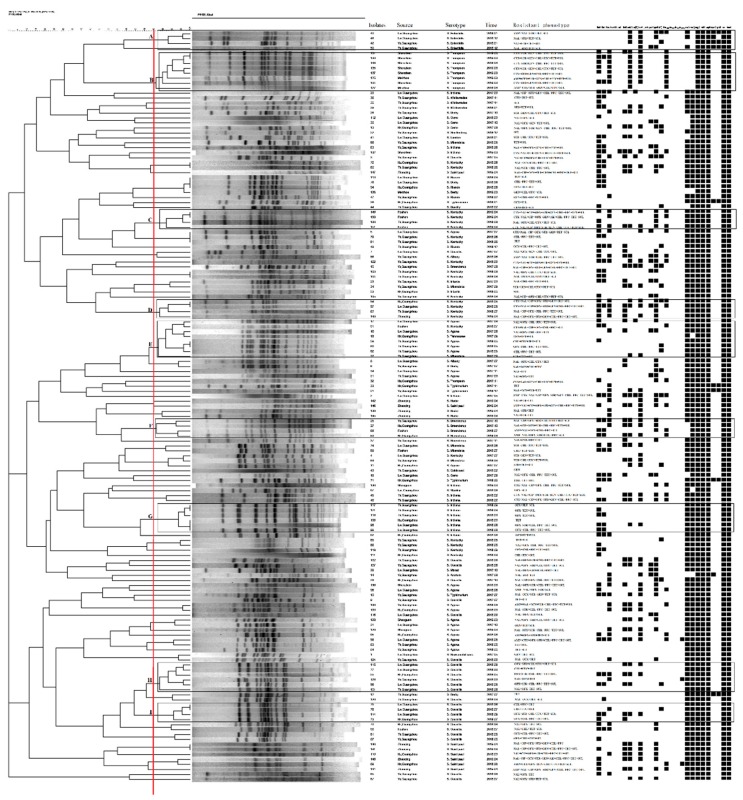
Dendrogram of PFGE patterns of 151 *Salmonella* isolates from retail duck meats in Southern China showing the antibiotic resistance phenotype, resistance genes and virulence gene patterns. A black box indicates carrier-related genes.

Based on ≥ 80% homology cutoff level, 136 PFGE pulsed-field electrophoretograms, cladograms, or patterns were observed among the strains with most of the strains grouping within 31 major clusters. There were 54 strains which comprised single cladograms. As shown in Figure 2, cluster B (8/151, 5.3%) and cluster E (8/151, 5.3%) were dominant, followed by clusters H (6/151, 4.0%) and G (6/151, 4.0%). It is worth noting that the 20 MDR *S*. Kentucky isolates were mainly distributed in clusters C (4/20, 20.0%) and D (4/20, 20.0%), which had similar quinolone-resistance phenotypes and resistance genes. In short, the majority of *Salmonella* isolates of the same serovars were clustered in the same PFGE clusters, such as A (*S.* Enteritidis), F (*S.* Braenderup) and G (S. Indiana). However, it was found that different serotypes could be included in the same PFGE cluster, such as E (5 *S*. Agona, 1 *S*. Kentucky, 1 *S*. Mbandaka and 1 *S*. Tennessee). A dendrogram of PFGE patterns is shown in Figure 2.

## 4. Discussion

Although the safety of retail duck meat has attracted consumers’ attention, very few studies have reported the presence of *Salmonella* in duck meat in China. In this study, we found high levels of *Salmonella* contamination in duck meat (41.4%) obtained from retail markets in in Southern China, which was higher than the prevalence rates of 27.3% [7], 23.5% [14], 27% [17], and 11.6% [32] reported in previous studies worldwide. These results show that *Salmonella* contamination in retail duck meat in Southern China is serious public health concern. At the same time, the prevalence (41.4%) of *Salmonella* contamination in duck meat identified in this study is higher than that reported in chicken meat in India, Malaysian, Vietnam, Egypt and the UK [18,23,25,27,33,34]. The differences in the prevalence of *Salmonella* contamination in these countries may be due to the differences in the breeding environment, the health and safety regulations of the retail market, meat quality variation, and the methods of isolation. In addition, we found that the *Salmonella* contamination was more common in summer, which may be closely related to the high temperature and humidity associated with the climate in summer in the subtropical region of Guangdong Province [35]. Thus, strategies to improve food safety should be implemented in subtropical areas, focusing on the need to strengthen supervision of retail markets during the summer months. Such strategies could include improving the market management system (stall sales, tool cleaning and regular disinfection) and ensuring high standards of environmental hygiene (retail stand clean and dry) to protect public health.

Twenty-six different *Salmonella* serovars were identified in retail duck meat and the distribution of the serotypes was very different from that in other regions. *S.* Typhimurium, *S*. Enteritidis and *S*. Indiana have been regarded as the most common duck meat serotypes [7,10,14,17,32]. However, the prevalent serotypes detected in our study were *S*. Corvallis, *S*. Kentucky and *S*. Agona, which is consistent with more recent reports worldwide [28,36,37,38,39]. Ciprofloxacin-resistant *S*. Kentucky, in particular, has attracted great attention worldwide [40,41]. In addition, *S*. Agona is one of the top 10 serotypes responsible for nosocomial infections in China [8]. The serotypes found in retail duck meat are also different from those reported in chicken meat worldwide [8,19,27,42,43,44]. This might be because the distribution of *Salmonella* serotypes is largely related to geographical location, sampling season and treatment methods [7]. Additionally, *S.* Bareilly and *S*. Molade have not previously been reported in Chinese food. Thus, the identification of these two *Salmonella* serotypes not only expands the *Salmonella* strain bank in China, but also provides a new basis for follow-up research. Interestingly, the prevalent serotypes of *Salmonella* in retail duck meat were similar to those reported in the previously retail chicken meat in Guangdong Province [8]. These results indicate that *S*. Corvallis, *S*. Kentucky and *S*. Agona are widely prevalent in animal foods in Southern China and serve as warning that these *Salmonella* serotypes have the potential to spread horizontally in different animal-derived foods, which may lead to public health problems.

MDR *Salmonella* was most commonly (133/151, 88.1%) detected antimicrobial resistance profile detected in retail duck meat, which may be the result of abuse of antibiotics in duck farming, especially as growth promoters and disease prevention [9,27]. MDR was widely distributed in various serotypes of *Salmonella* in this study, especially in *S*. Kentucky and *S*. Indiana. We found that 72.7% of *S*. Kentucky isolates were resistant to six or more antimicrobial agents, which is a much higher percentage that of the other serotypes. Our results highlight the serious issue of *Salmonella* multidrug resistance in retail duck meat in South China, which could result in the evolution of *Salmonella* into a super bacteria and risk to public health [45,46]. Therefore, the strengthened and continuous surveillance of antimicrobial resistance in different serotypes of *Salmonella* is conducive to the further understanding of MDR *Salmonella* and safeguarding consumer health.

It is noteworthy that we detected 12 (7.9%) *Salmonella* isolates (7 *S*. Kentucky, 4 *S*. Indiana and 1 *S*. Agona) that were resistant to both ciprofloxacin and cefotaxime, which is similar to other reports from China [8] and India [27]. Thus, these findings highlight the requirement for greater vigilance, especially for ciprofloxacin-resistant *S*. Kentucky, which represents a serious threat to public health [40,41]. In addition, the patterns of antimicrobial resistance genes were basically consistent with the antibiotic resistance phenotypes. However, the quinolone- and cephalosporin-related resistance genes detected are somewhat different from those reported previously [27,28,46,47,48,49]. This discrepancy may be due to the large number of related genes that mediate resistance to these two drugs or alternatively, due to variations in the mechanism of *Salmonella* resistance as a result of geographical or other factors, which need further investigation. We also found that *Salmonella* isolates with different serotypes showed variations in virulence-associated genes, which indicates an association between serotypes and potential pathogenicity [50]. Therefore, strategies to control different *Salmonella* serotypes that may be pathogenic are required to protect public health.

A total of 136 PFGE genotypes were identified in 151 *Salmonella* isolates, which provides an important overview of *Salmonella* genetic diversity. PFGE genotypes allowed grouping of the majority of isolates according to *Salmonella* serotype, which was consistent with the grouping reported previously [31,51]. PFGE data showed that the dominant cluster B was composed of MDR *S.* Thompson isolates from different cities. This was followed by cluster H composed of MDR *S*. Corvallis isolates from different seasons. In addition, these isolates exhibited similar resistance phenotypes, resistance genes and virulence genes patterns. These data indicate these MDR *Salmonella* isolates from different cities and seasons have similar genetic relationships, with the potential of cloning and transverse transmission, thus representing a public health risk [16,50,52]. We also found that different serotypes can be included in the same PFGE cluster, showing that these different serotypes of *Salmonella* are closely related and deserve further study.

## 5. Conclusions

In summary, we examined the *Salmonella* epidemiology and antimicrobial resistance isolates obtained from duck meat samples in retail markets in Southern China. Our findings show the high prevalence and extent of serotype diversity within *Salmonella* isolated from duck meat samples and *S*. Corvallis, *S*. Kentucky and *S*. Agona were the most common serovars among isolates. Notably, the prevalence of MDR *S*. Kentucky and *S*. Indiana carrying multiple virulence-associated genes indicates the potential risk of *Salmonella* foodborne infections. Isolates from different districts had similar PFGE patterns indicating that circulating foodborne *Salmonella* constitutes a potential public health issue across different districts.

## Figures and Tables

**Table 1:** Prevalence of *Salmonella* isolated from duck meats at retail markets in Southern China.

Season of Isolation	Samples (*n*)	Number Positive	Ratio (%)
Spring	140	46	32.9
Summer	85	53	62.4
Autumn	80	37	46.3
Winter	60	15	25.0
Total	365	151	41.4

**Table 2:** Antimicrobial resistance phenotypes of *Salmonella* isolates (*n* = 151).

Antibiotics ^1^	Minimum Inhibitory Concentration (μg/mL) Distribution of 151 *Salmonella* isolates														
	≤0.125	0.25	0.5	1	2	4	8	16	32	64	128	256	≥512	Resistant Breakpoint	Resistance % (*n* = 151)
AMP					91	9	14	23	12	1	1			≥32	9.3(*n* = 14)
FEP	64	50	2	1	19	2	8	5						≥32	0
CTX	119	1	3	3			2	6	8	9				≥4	16.6(*n* = 25)
IPM		31	32	79	9									≥4	0
NAL						10	27	32	33	8	16	23	2	≥32	53.6(*n* = 81)
CIP	21	3	23	34	41	5	9	3	9	3				≥4	19.2(*n* = 29)
OFX	16	3	32	14	29	25	11	8	3	7	3			≥2	56.9(*n* = 86)
STR						9	33	35	6	10	35	4	19	≥64	45.0(*n* = 68)
GEN			11	40	9		44	18	1	28				≥16	31.1(*n* = 47)
AMK				12	120	8	6	1	2	2				≥64	1.3(*n* = 2)
CHL				1		53	2	1		3	69	19	3	≥32	62.3(*n* = 94)
FFC					6	44	10	1	1	1	61	24	3	≥16	60.3(*n* = 91)
TET						22			4	58	67			≥16	85.4(*n* = 129)
SUL										2	6	16	127	≥512	84.1(*n* = 127)
PB		10	24	105	10	2								≥ 8	0

^1^ AMP (ampicillin), FEP (cefepime), CTX (cefotaxime), IPM (imipenem), NAL (nalidixic acid), CIP (ciprofloxacin), OFX (ofloxacin), STR (streptomycin), GEN (gentamicin), AMK (amikacin), CHL (chloramphenicol), FFC (florfenicol), TET (tetracycline), SUL (sulfadiazine), PB (polymyxin B).

**Table 3:** Multidrug resistance (MDR) observed among *Salmonella* serotypes (*n* = 151).

Serotype (No. Tested)	Number of Resistant Isolates to Indicated Number of Antimicrobials (%)				
	0–2	3–5	6–8	9–11	Total (Resistance ≥ 3)
*S*. Corvallis (25)	1(4.0)	15(60.0)	9(36.0)		24(96.0)
*S*. Kentucky (22)	2(9.1)	4(18.2)	10(45.5)	6(27.3)	20(90.9)
*S*. Agona (20)	3(15.0)	8(40.0)	8(40.0)	1(5.0)	17(85.0)
*S*. Indiana (14)	1(7.1)	5(35.7)	4(28.6)	4(28.6)	13(92.9)
*S*. Thompson (8)					8(100)
*S*. Saint Paul (8)			3(37.5)	5(62.5)	8(100)
*S*. Typhimurium (6)	2(33.3)	2(33.3)	2(33.3)		4(66.7)
*S*. Mbandaka (6)	1(16.7)	4(66.7)	1(16.7)		5(83.3)
*S*. Braenderup (6)		1(16.7)	3(50.0)	2(33.3)	6(100)
*S*. Derby (5)	1(20.0)	3(60.0)	1(20.0)		4(80.0)
*S*. Cerro (4)		1(25.0)	3(75.0)		4(100)
*S*. Rissen (4)	2(50.0)	2(50.0)			2(50.0)
*S*. Enteritidis (4)		4(100)			4(100)
Others ^2^ (19)	5(26.3)	12(63.2)	2(10.5)		14(73.7)
Total (151)	18(11.9)	61(40.1)	54(35.8)	18(11.9)	133(88.1)

^2^ Others included strains for which ≤ 3 of each serotype were tested, e.g., *S*. Weltevreden, Hadar, Infantis, Albany, Tennessee, Stanley, Senftenberg, London, Anatum, Bovismorbificans, Goldcoast. Bareilly and *S*. Molade.

**Table 4:** Distribution of antibiotic resistance genes among *Salmonella* isolates.

Antimicrobial Classes	No. of Resistant Isolates	Genes Detected	No. of Isolates (%)
Sulfonamides	127	*Sul I*	80 (63.0)
		*Sul II*	41 (32.3)
Tetracyclines	129	*tet A*	62 (48.1)
		*tet B*	
Chloramphenicols	94	*cat1*	1 (1.1)
		*floR*	53 (56.4)
Aminoglycosides	68	*aadA1*	57 (83.8)
		*Aaca(3)-Ia*	
		*strA*	64 (94.1)
Fluoroquinolones	81	*qnrA*	
		*qnrB*	18 (22.2)
		*qnrS*	64 (79.0)
		*aac (6′)-Ib*	21 (25.9)
β-lactamase	34 ^3^	*bla _TEM_*	34 (100)
		*bla _PSE_*	
		*bla _SHV_*	
		*bla _CTX-M_*	3 (8.8)

^3^ The 34 *Salmonella* isolates including those resistant to ampicillin (14) or resistant to cefotaxime (25).

**Table 5:** Distribution of virulence genes among *Salmonella* isolates with different serovars.

Serotype (No. Tested)	Number of Virulence Genes Positive Isolates (%)								
	*avrA*	*ssaQ*	*mgtC*	*siiD*	*sopB*	*spvC*	*spvR*	*stn*	*fimA*
*S*. Corvallis (25)	25(100)	25(100)	25(100)	25(100)	25(100)			25(100)	25(100)
*S*. Kentucky (22)	22(100)	22(100)	22(100)	22(100)	22(100)			22(100)	22(100)
*S*. Agona (20)	20(100)	20(100)	20(100)	20(100)	20(100)			20(100)	20(100)
*S*. Indiana (14)	14(100)	14(100)	14(100)	14(100)	14(100)			14(100)	14(100)
*S*. Thompson (8)			8(100)	8(100)	8(100)			8(100)	8(100)
*S*. Saint Paul (8)	8(100)	8(100)	8(100)	8(100)	8(100)			8(100)	8(100)
*S*. Typhimurium (6)	6(100)	6(100)	6(100)	6(100)	6(100)	4(66.7)	4(66.7)	6(100)	6(100)
*S*. Mbandaka (6)	6(100)	6(100)	6(100)	6(100)	6(100)			6(100)	6(100)
*S*. Braenderup (6)	3(50.0)	6(100)	6(100)	6(100)	6(100)			6(100)	6(100)
*S*. Derby (5)	4(80.0)	4(80.0)	5(100)	5(100)	5(100)	1(20.0)	1(20.0)	5(100)	5(100)
*S*. Cerro (4)		4(100)	4(100)	4(100)	4(100)			4(100)	4(100)
*S*. Rissen (4)	4(100)	4(100)	4(100)	4(100)	4(100)			4(100)	4(100)
*S*. Enteritidis (4)	3(75.0)		4(100)	4(100)	4(100)	4(100)	3(75.0)	4(100)	4(100)
Others ^4^ (19)	16(84.2)	16(84.2)	19(100)	19(100)	19(100)	1(5.3)		19(100)	19(100)
Total (151)	131(86.8)	135(89.4)	151(100)	151(100)	151(100)	10(6.7)	8(5.3)	151(100)	151(100)

^4^ Others, see Table 3.

## Supplementary Materials

The following are available online at https://www.mdpi.com/2076-2607/8/3/444/s1, Table S1. Primers for amplification of resistance genes used in this study, Table S2. Primers for amplification of virulence genes used in this study.
