# Supplementary material for: Prevalence, Antimicrobial Resistance, Virulence Genes and Genetic Diversity of Salmonella Isolated from Retail Duck Meat in Southern China
Source: Microorganisms. 2020 Mar 21;8(3):444. doi: 10.3390/microorganisms8030444 (PMC7143943; doi:10.3390/microorganisms8030444)
Supplement: Supplementary file 1 [file microorganisms-08-00444-s001.pdf]

Table S1. Primers for amplification of resistance genes used in this study.

Antimicrobial classes	Gene	Primer sequences (5′-3′)	Size (bp)	Reference
Sulfonamides	*sul I* -F	TGCAGGCTGGTGGTGGTTA	425	[1]
	sul I -R	CGCGTGGGTGCGGACGT		
	sul II -F	CATTCCCGTCTCGCTCGA	435	[1]
	sul II -R	GCGCGCAGAAAGGATTT		
Tetracyclines	*tetA*-F	TCGCTTGCCGCATTT	474	[1]
	tetA-R	CGCGTATAGCTTGCCG		
	tetB-F	GACACTCTATCATTGAT	571	[1]
	tetB-R	GACAATATTTAGCAACG		
Chloramphenicols	*cat1*-F	AGTGGAATAACGAACGAGC	470	[2]
	cat1-R	TCAGCAAGCGATATACGCAG		
	floR-F	CTGAGGGTGTCGTCATCTAC	637	[2]
	floR-R	GCTCCGACAATGCTGACTAT		
Aminoglycosides	*aadA1*-F	TTTGCTGGTTACGGTGAC	497	[3]
	aadA1-R	GCTCCATTGCCCAGTCG		
	Aaca(3)-Ia-F	GCTCCATTGCCCAGTCG	436	[1]
	Aaca(3)-Ia-R	TGAGGGCTGCTCTTGATCTT		
	strA-F	CCAATCGCAGATAGAAGGC	548	[3]
	strA-R	ATCGTCAAGGGATTGAAACC		
Fluoroquinolones	*qnrA*-F	AGAGGATTTCTCACGCCAGG	580	[4]
	qnrA-R	TGCCAGGCACAGATCTTGAC		
	qnrB-F	GGMATHGAAATTCGCCACTG	264	[4]
	qnrB-R	TTTGCYGYYCGCCAGTCGAA		
	qnrS-F	GCAAGTTCATTGAACAGGGT	428	[4]
	qnrS-R	TCTAAACCGTCGAGTTCGGCG		
	aac (6')-Ib-F	TTGCGATGCTCTATGAGTGGCTA	482	[4]
	aac (6')-Ib-R	CTCGAATGCCTGGCGTGTTT		
β-Lactams	*bla* $_{TEM}$-F	ATGAGTATTCAACATTTCCG	964	[5]
	bla $_{TEM}$-R	ACCAATGCTTAATCAGTGAG		
	bla $_{PSE}$-F	AATGGCAATCAGCGCTTCCC	598	[5]
	bla $_{PSE}$-R	GGGGCTTGATGCTCACTACA		
	bla $_{SHV}$-F	TTCGCCTGTGTATTATCTCCCTG	854	[5]
	bla $_{SHV}$-R	TTAGCGTTGCCAGTGCTCG		
	bla $_{CTX-M}$-F	TTTGCGATGTGCAGTACCAGTAA	544	[6]
	bla $_{CTX-M}$-R	CGATATCGTTGGTGGTGCCATA		

Table S2. Primers for amplification of virulence genes used in this study.

Gene	Location	Primers sequence（5′—3′）	Size (bp)	Reference
avrA	SPI-1	F: CCTGTATTGTTGAGCGTCTGG<br>R: AGAAGAGCTTCGTTGAATGTCC	422	[7]
ssaQ	SPI-2	F: GAATAGCGAATGAAGAGCGTCC<br>R: CATCGTGTTATCCTCTGTCAGC	455	[7]
mgtC	SPI-3	F: TGACTATCAATGCTCCAGTGAAT<br>R: ATTTACTGGCCGCTATGCTGTTG	677	[7]
siiD	SPI-4	F: GAATAGAAGACAAAGCGATCATC<br>R: GCTTTGTCCACGCCTTTCATC	655	[7]
sopB	SPI-5	F: TCAGAAGGCGTCTTACCACTC<br>R: TACCGTCCTCATGCACACTC	1231	[7]
spvC	Plasmid	F: CCATCTACAAATAAGCACCTGA<br>R: CATTTCGCCACCATCACG	597	[8]
spvR	Plasmid	F: GGCACTCTTATCCCAACCG<br>R: TAACATCGCCAGCCCTTG	555	[8]
stn	Enterotoxin	F: ATTGAGCGCTTTAATCTCCT<br>R: GCTGTTGAATCTGTACCTGA′	543	[2]
fimA	Fimbrial	F: CACTAAATCCGCCGATCAAACG<br>R: AAAGGTGGCGTCGGCATTA	394	[8]

References

1. Zhu, A.; Zhi, W.; Qiu, Y.; Wei, L.; Tian, J.; Pan, Z.; Kang, X.; Gu, W.; Duan, L. Surveillance study of the prevalence and antimicrobial resistance of *Salmonella* in pork from open markets in Xuzhou, China. *Food Control* **2019**, *98*, 474–480, doi:10.1016/j.foodcont.2018.07.035.
2. Li, K.; Ye, S.; Alali, W.Q.; Wang, Y.; Wang, X.; Xia, X.; Yang, B. Antimicrobial susceptibility, virulence gene and pulsed-field gel electrophoresis profiles of *Salmonella enterica* serovar Typhimurium recovered from retail raw chickens, China. *Food Control* **2017**, *72*, 36–42, doi:10.1016/j.foodcont.2016.07.032.
3. Abatcha, M.G.; Effarizah, M.E.; Rusul, G. Prevalence, antimicrobial resistance, resistance genes and class 1 integrons of *Salmonella* serovars in leafy vegetables, chicken carcasses and related processing environments in Malaysian fresh food markets. *Food Control* **2018**, *91*, 170–180, doi:10.1016/j.foodcont.2018.02.039.
4. Yang, B.; Qiao, L.; Zhang, X.; Cui, Y.; Xia, X.; Cui, S.; Wang, X.; Meng, X.; Ge, W.; Shi, X., et al. Serotyping, antimicrobial susceptibility, pulse field gel electrophoresis analysis of *Salmonella* isolates from retail foods in Henan Province, China. *Food Control* **2013**, *32*, 228–235, doi:10.1016/j.foodcont.2012.11.022.
5. Zhang, Z.; Cao, C.; Liu, B.; Xu, X.; Yan, Y.; Cui, S.; Chen, S.; Meng, J.; Yang, B. Comparative Study on Antibiotic Resistance and DNA Profiles of *Salmonella enterica* Serovar Typhimurium Isolated from Humans, Retail Foods, and the Environment in Shanghai, China. *Foodborne Pathog. Dis.* **2018**, *15*, 481–488, doi:10.1089/fpd.2017.2414.
6. Zhu, Y.; Lai, H.; Zou, L.; Yin, S.; Wang, C.; Han, X.; Xia, X.; Hu, K.; He, L.; Zhou, K., et al. Antimicrobial resistance and resistance genes in *Salmonella* strains isolated from broiler chickens along the slaughtering process in China. *Int. J. Food Microbiol.* **2017**, *259*, 43–51, doi:10.1016/j.ijfoodmicro.2017.07.023.
7. Huehn, S.; La Ragione, R.M.; Anjum, M.; Saunders, M.; Woodward, M.J.; Bunge, C.; Helmuth, R.; Hauser, E.; Guerra, B.; Beutlich, J., et al. Virulotyping and antimicrobial resistance typing of *Salmonella enterica* serovars relevant to human health in Europe. *Foodborne Pathog. Dis.* **2010**, *7*, 523–535, doi:10.1089/fpd.2009.0447.
8. Qiao, J.; Zhang, Q.; Alali, W.Q.; Wang, J.; Meng, L.; Xiao, Y.; Yang, H.; Chen, S.; Cui, S.; Yang, B. Characterization of extended-spectrum β-lactamases (ESBLs)-producing *Salmonella* in retail raw chicken carcasses. *Int. J. Food Microbiol.* **2017**, *248*, 72–81, doi:10.1016/j.ijfoodmicro.2017.02.016.
